# Edwin Ellen Goldmann (1862–1913)

**DOI:** 10.1007/s00415-023-11668-4

**Published:** 2023-03-28

**Authors:** Rüdiger Adam, Axel C. Hüntelmann

**Affiliations:** 1 University Children’s Hospital, Medical Faculty Mannheim, Heidelberg University, Mannheim, Germany; 2 Institute for the History and Ethics of Medicine, Charité—University Medicine Berlin, Berlin, Germany

No scholarly article on the history of the blood–brain barrier may overlook Edwin Ellen Goldmann’s (Fig. 1) work; scientists working in the field are familiar with his seminal experiments, especially regarding aspects of the blood–CSF barrier and choroid plexus function.

**Fig. 1:**
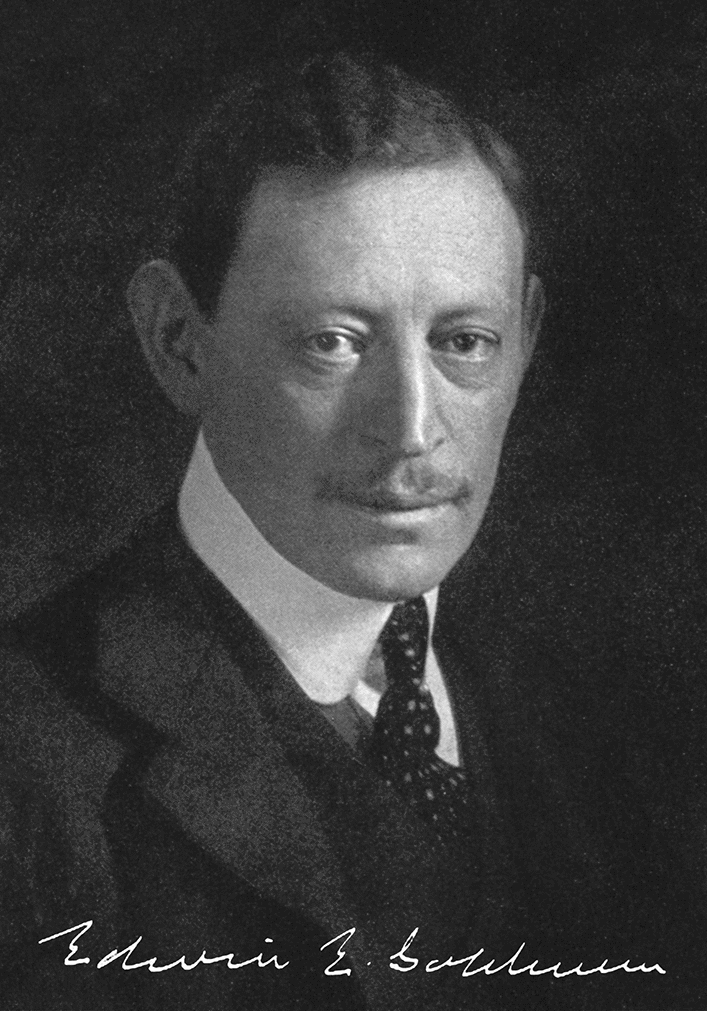
Edwin E. Goldmann (1862–1913). Galerie hervorragender Ärzte und Naturforscher, Blatt 333. Beilage zu Münchener Medizinische Wochenschrift, München, J. F. Lehmann Verlag, 1913, with slight graphical modifications;  property of RA

Goldmann was born in 1862, one of five children of German–Jewish immigrants, in Burgersdorp, South Africa. He was brought up in a respected bourgeois family, interested in arts, music, and biology [9]. When he was 14 years old, his family returned to Europe and settled in Breslau. After graduating from Gymnasium, Goldmann studied medicine at the University of Freiburg. He obtained his MD in 1886 and a year later presented his dissertation on cystinuria and sulfur excretion in the urine (Experimentelle Beiträge zur Lehre von der Cystinurie und der Schwefel-Ausscheidung im Harne, Freiburg, 1886), supervised by Eugen Baumann, chair of the faculty of medical chemistry at Freiburg.

After his biochemical beginnings, Goldmann moved in 1888 to the renowned Institute of Anatomical Pathology of the Senckenberg Foundation in Frankfurt on Main to become a “first-rate microscopist” [9]. In Frankfurt, he worked with the Institute’s director, Carl Weigert, a cousin of Paul Ehrlich and a pioneer in the development of histological staining techniques, especially for neuroglia and myelin.

Following his histopathological training at Weigert’s Institute, Goldmann commenced his practical medical career. In 1888, he became an assistant at the University Surgical Hospital in Freiburg where he remained for 10 years, simultaneously pursuing a clinical and an academic career. In 1891, he qualified as *Privatdozent* with a thesis on neuromata, applying the Weigert method of myelin staining [6]. In 1895, he was appointed Professor extraordinarius. Goldmann left the University in 1898 to head the newly founded Deaconess Hospital in Freiburg as chief physician. He remained there until his death in 1913.

During his time as a surgeon in Freiburg, Goldmann published numerous articles on a wide spectrum of clinical and pathoanatomical topics: wound treatment and skin transplantation, treatment of lymphoma and other tumors, extremity malformations and hip dislocations, gynecological operations and urethral strictures, and infectious diseases, including diphtheria and tuberculosis.

Apart from clinical research, Goldmann also remained profoundly interested in fundamental histopathological research throughout his life. During his Freiburg years, he stayed in close contact with Paul Ehrlich. Ehrlich had revolutionized the visualization of biological structures and the interrelationship of cells and organs by developing a method of “intravital staining” that injected dyes into living animals and then examined their organs histopathologically [3].

Ehrlich and Goldmann exchanged literature and discussed scientific theories, research practices, and strategies for disseminating their work. Goldmann also received high-quality dyes that were otherwise difficult to obtain, as well as laboratory animals, and even financial support [4]. This intense collaboration saw Goldmann becoming one of the first scientists to systematically apply and further develop Ehrlich’s histopathological techniques of intravital staining.

In his previous experiments, when he had parentally injected certain dyes into various vertebrates, Ehrlich did observe a selective lack of staining of the brain. He attributed this observation to a lack of affinity between brain tissue and the dyes, and argued against the hypothesis that the CNS barrier was based on vascular properties, as proposed by other researchers at the time [2, 5]. As a result, Ehrlich has been described as the “reluctant discoverer of the blood–brain barrier phenomenon” [10].

Goldmann elegantly pursued his mentor’s intravital stain studies in numerous series of experiments. By systemic injections of predominantly trypan blue into animals of diverse species he demonstrated that virtually all tissues of the challenged animal turned blue, except the CNS. He stated that the brain and spinal cord remained snow-white, while the rest of the body, including the plexus choroidei, stained intensely. He later wrote that he was puzzled “that large quantities of dye, which circulate in the cerebral vessels during intravenous application, are not able to penetrate even the finest capillary wall” [7, 8].

Goldmann also conducted what could be characterized as *counter-experiments*: he injected trypan blue directly into the subarachnoid space of animals either in the lumbar sac or the cisterna magna, thereby staining most of and only the CNS. He thus astutely refuted Ehrlich’s affinity theory, and demonstrated, on the one hand, that the brain was physiologically separate from the rest of the body, and that, on the other hand, no relevant barrier existed between the CSF and the brain parenchyma (a *CSF-brain barrier*) [1, 7, 8]. Today, neuroanatomists still refer to these two experiments, the first administering the dye systemically, the second intrathecally, as the “first and second Goldmann experiments”.

Goldmann also observed that the choroid plexus epithelial cells were responsible for keeping intravital stains from entering the CSF. He characterized the role of the choroid plexus as a placenta-like physiological barrier membrane (*physiologische Grenzmembran*). Accordingly, as in the case of the placenta, he spoke of a “physiological limiting membrane” in the nervous system, the effectiveness of which was based on the vital cellular mechanism of the plexus epithelium. “It is not special vascular facilities or vascular alterations of the meninges that prevent or promote the passage of blood-soluble substances in the norm, but the respective physiological or pathological condition of the plexus cells” [8].

Although famous for his illustrative description and clear visualization of the existence of a blood–brain barrier phenomenon, Goldmann cannot be credited with being the first scientist to publish about it. However, he provided convincing evidence of a compartimentalization of the brain and the rest of the body, and he can be credited for carrying out the first systematic experiments on the principle of the blood–CSF barrier [10].

Unfortunately, Goldmann tragically suffered from cancer and was unable to complete his opus magnum as he had already foreshadowed in his latest work on the blood–brain barrier and plexus [8]. “With this splendid man science loses one of her best representatives”, lamented Ehrlich in *The London Times* after Goldmann passed away in 1913.

## References

[1] Bentivoglio M, Kristensson K (2014). Tryps and trips: cell trafficking across the 100-year-old blood-brain barrier. Trends Neurosci.

[2] Biedl A, Kraus R (1898). Über eine bisher unbekannte toxische Wirkung der Gallensäuren auf das Centralnervensystem. Centralblatt Innere Medizin.

[3] Ehrlich P (1885). Das Sauerstoff-Bedürfniss des Organismus, eine farbenanalytische Studie.

[4] Ehrlich P (1908) Paul Ehrlich an E. Goldmann, 12.11.1908. Rockefeller Archive Center, Paul Ehrlich Collection

[5] Ehrlich P (1902) Ueber die Beziehungen von chemischer Constitution, Vertheilung und pharmakologischer Wirkung: Vortrag, gehalten im Verein für Innere Medicin am 12. December 1898. Internationale Beiträge zur Inneren Medicin. Ernst von Leyden zur Feier seines 70jährigen Geburtstages. August Hirschwald, Berlin

[6] Goldmann EE (1893). Beitrag zu der Lehre von den Neuromen. Beitr Klin Chir.

[7] Goldmann EE (1909). Die äussere und innere Sekretion des gesunden und kranken Organismus im Lichte der "vitalen Färbung". Beitr Klin Chir.

[8] Goldmann EE (1913). Vitalfärbung am Zentralnervensystem. Abhandlungen der Königlich Preussischen Akademie der Wissenschaften, Physikalisch-Mathematische Classe.

[9] Obituary, (1913). Edwin Ellen Goldmann, M.D. Freiburg. Honorary Ordinarius Professor of Surgery at the University of Freiburg in Baden. The Lancet.

[10] Saunders NR, Dreifuss JJ, Dziegielewska KM, Johansson PA, Habgood MD, Møllgård K, Bauer HC (2014). The rights and wrongs of blood-brain barrier permeability studies: a walk through 100 years of history. Front Neurosci.

